# The Encouragement and Management of Breast-Feeding

**Published:** 1913-08-23

**Authors:** 


### MATERNITY AND MOTHERING.

(Inquiries and Correspondence are invited.)

### The Encouragement and Management of Breast-Feeding.

The strenuous preaching of the gospel of maternal nursing for infants, to which the medical profession has long devoted itself, is beginning to show some return for the time and energy expended on it. Among industrial workers economic conditions are slowly improving, and it is such improvement that alone, with education, can combat the evils of bottle-feeding as they are seen in the labouring classes. In the middle and upper classes there is now a widespread appreciation of the advantages of breast-feeding, and desire to secure them. There are, of course, still many mothers too selfish or careless to want to nurse their babies; but not nearly so many in proportion as there were a few years ago. So that, in general terms, it is probably true that the deplorably low percentage of properly nurtured infants in this country is steadily, if slowly, increasing, and likely to increase.

There are, however, many mothers who, with every wish to do their duty to their children, find themselves discouraged by failure, or apparent failure, in the attempt. Not at all infrequently all that is needed is expert advice, which will speedily show how matters can be put right; but, unfortunately, good advice from a person qualified to give it is often not asked, and instead the conclusion is jumped at that the milk is too poor, or too strong, or too scanty, or does not agree with the child, and a change is quite unnecessarily made to bottle-feeding. It must be admitted, also, that advice in this latter sense is sometimes tendered by medical men who do not always take enough care to probe thoroughly the system of feeds and to ascertain what needs correction.

### An Expert Symposium.

With these premisses we would direct attention to three peculiarly interesting and valuable papers upon various aspects of breast-feeding which have recently been written by Dr. Forsyth, Dr. Lucy Naish, and Dr. Cran respectively.* There is still a great deal to be done before the function of lactation will be fully elucidated; and each of these papers contains substantial contributions to the subject.

The communications of Dr. Forsyth and Dr. Cran are concerned largely with the question of the quantity of maternal milk which an infant does, or ought to, get at each feed from the breast. The French obstetricians, following the views of Budin, have for some years been preaching and practising a system of strict regulation of the quantity of maternal milk to be taken at each feed. This system involves the perpetual use of the weighing machine before and after feeding; and Dr. Variot, of Paris, whose views are adopted and reported by Dr. Cran, protests very strongly against the method on the ground that it has led to hypo-alimentation, or in plain English chronic starvation. Budin regulates the quantity of food allowed to the infant according to its body weight. For children of five or six months, to give an example, he allows 100 gm. of milk in twenty-four hours per kilogram of body weight. He is very emphatic on the evils of over-feeding, and his teaching seems to have resulted in a veritable tyranny of the weighing machine. This system, which is very prevalent in France, Variot condemns roundly. The normal new-born child, he maintains, needs one-sixth, not one-tenth, of its own body weight in milk per day.

### Infant Feeding and the Scales.

In this country the systematic weighing of breast-fed babies before and after (and during) each meal is not merely not general, but is very nearly unknown. Budin's doctrines have certainly no adherents, because very few doctors have ever heard of them. So much is this the case that Dr. Forsyth, who carried out prolonged observations upon the quantity of milk obtained from the mother by a young infant, is practically breaking new ground in this country. His results, as he notes, have to be received with some caution, because the case was an isolated one, and may not be typical of the average baby. Thus, although the weekly consumption of milk did show a general tendency to increase with the increasing size and weight of the infant, the increase was neither regular nor uniform: during the second week of life an actually greater quantity of milk was taken than during either the third or fourth week. Moreover, the daily consumption during each separate week was frequently very different indeed from the average: there were differences of as much as 60 per cent. observed between separate daily quantities in one and the same week.

### Unexplained Fluctuations.

In spite of these extraordinary fluctuations, which appeared to be quite fortuitous and did not correspond to any observed departures from the normal in either mother or child, the gain in body weight (of the infant) progressed steadily and regularly. A chart reproduced in Dr. Forsyth's article shows very clearly the contrast between the weight line and the irregular fluctuating line, which represents the quantity of milk consumed by the infant.

Individual feeds varied even more widely still. Thus in the first week the largest feed was two ounces, the smallest a quarter of an ounce. In the second week the maximum was four and a half ounces (an enormous quantity for so young a child), and the smallest a quarter of an ounce. In the fourth week the largest feed was three and a half ounces, the smallest half an ounce: in the seventh, six and a half ounces and one and a quarter ounce respectively.

Dr. Forsyth confesses himself unable to account for these variations. On the whole he inclines to

* *The Lancet*, June 14, 1913, pp. 1656-1660.

place the responsibility for them upon the mother rather than on the child. But he does not dogmatise on what factors affect her, nor how to control them. "Whether she is fed generously or moderately, drinks much milk or little, takes or refrains from oatmeal stout and other reputed galactagogues, no reasonably scientific proof is yet forthcoming that these measures influence the activity of the mammary glands."

### A Pound of Practice.

The third of the papers constituting this symposium is of a different, but not less meritorious, type. It is by Dr. Lucy Naish, who has not merely had plenty of experience in advising mothers in London and Sheffield, but has also herself nursed her six children; and it deals with innumerable practical details of the management (and mismanagement) of the function of lactation. Personal experience is evident throughout this contribution; the many wrinkles and hints which it contains are just those all-important matters of detail which may make all the difference between success and failure in maternal nursing, and so between a thriving and a peaked baby.

First of all, Dr. Naish lays stress upon the weak and aching sensations of the new-made mother, whose prime necessities for the first two days are rest and sleep. If circumstances make it difficult for her to relinquish all household worries at this time, her rest is broken and her vigour will not return as rapidly as it otherwise would. At this period there is normally very little secretion of milk, and the baby frequently cries a good deal. The young mother draws the conclusion that the child is hungry, and worries because she cannot provide it with sufficient food; whereas in fact the child's crying is not (as a rule) that of hunger but more often of thirst, and to be assuaged by a teaspoonful of plain boiled water. The mother's anxieties on this point should be soothed away by assurances that all is well and that the child is not hungry.

### Infantile Peculiarities.

The curious predilections of many babies for one particular breast also receive notice. Many babies will make every effort to refuse one breast, though they will greedily take the other, for no obvious reason. Firmness on the part of the mother and nurse will speedily cure this remarkable peculiarity. Sometimes a baby seems to have no inclination to suck the nipple at all, screaming and struggling for several minutes at a time before it will settle down to a meal. This is always liable to get on the nerves of an inexperienced mother, but should not be admitted as a reasonable excuse for discontinuing nursing the child. Cracked nipples, again, as every doctor knows, are a fertile deterrent of maternal nursing. Prevention is, of course, infinitely superior to cure; Mrs. Naish condemns the still popular method of hardening the skin during pregnancy with eau-de-Cologne or other alcoholic applications, and we quite agree with her. This is a point on which authorities differ, and many recognised text-books recommend the practice which Mrs. Naish denounces. With her, we vastly prefer the use of oily or fatty applications, if any are necessary: and this opinion is based upon experience and reflection.

### The Importance of Regularity.

Another point well enough known to medical men is the production of after-pains whenever the baby is put to the breast during the first few days of the puerperium. These pains are sometimes very severe, and mothers may then be led to think that something is wrong. In point of fact, such pains are extremely beneficial, inasmuch as they are due to firm contractions of the uterus; and much apprehension can be relieved if only this point is made clear to the patient by the doctor. The importance of absolute regularity in regard to the times at which the infant shall be offered the breast is also well emphasised by Dr. Naish. It is true that quite tiny babies often sleep very soundly, and are with difficulty waked when meal-times come round; but there is little doubt that it is right thus to arouse them, and to make them take their feeds at uniform intervals. At any rate, insistence on such regularity does conduce to a plentiful milk supply of good quality. In common with Dr. Forsyth, Dr. Naish finds there is no relation between the amount of milk which a given baby takes and the occurrence of crying afterwards. An occasional fit of crying after a breast feed should certainly not be regarded as evidence in favour of weaning.

### The Hygiene of the State of Lactation.

There are many more points in connection with lactation dealt with in Mrs. Naish's valuable paper, some of which have been dealt with in previous articles of this series. At present we have space to mention only two of them. One is her plea for plenty of walking exercise, fresh air, and sleep for the nursing mother, together with avoidance of over-feeding and of too great indulgence in milk. This latter recommendation is contrary to current beliefs, and we are not sure that in this matter Mrs. Naish is altogether right. The other is her conviction that there are special epochs at which the supply of breast-milk is especially apt to fail, when every precaution against this should be redoubled. These critical times are, she says, the ninth day, the third week, the sixth week, and the beginnings of the third and fourth months. We listen with respect to anything Dr. Naish has to say on this subject, but we must confess she advances no proof in favour of this hypothesis, nor any explanation of it. It may be true, but it is certainly not proved so far. The most valuable of Mrs. Naish's hints are, we believe, those concerned with the mental attitude of the mother. Beyond all doubt the lactating mother should lead a placid life of quietude and freedom from worry—the life of a cow, as the writer has heard one of his patients express it—or else the supply and quality of the mammary secretion will suffer.

